# Clinical outcomes of endoscopic submucosal dissection for large pedunculated colorectal carcinoma: A retrospective multicenter study

**DOI:** 10.1002/deo2.277

**Published:** 2023-08-13

**Authors:** Katsuaki Inagaki, Ken Yamashita, Shiro Oka, Fumiaki Tanino, Noriko Yamamoto, Yuki Kamigaichi, Hirosato Tamari, Tomoyuki Nishimura, Yuki Okamoto, Hidenori Tanaka, Takahiro Kotachi, Ryo Yuge, Yuji Urabe, Yasuhiko Kitadai, Shinji Tanaka

**Affiliations:** ^1^ Department of Gastroenterology Hiroshima University Hospital Hiroshima Japan; ^2^ Department of Endoscopy Hiroshima University Hospital Hiroshima Japan; ^3^ Division of Regeneration and Medicine Center for Translational and Clinical Research Hiroshima University Hospital Hiroshima Japan; ^4^ Department of Health Sciences Prefectural University of Hiroshima Hiroshima Japan

**Keywords:** colorectal neoplasms, complications, endoscopic submucosal dissection, large pedunculated colorectal carcinoma, treatment outcome

## Abstract

**Objectives:**

Complete en‐bloc resection of pedunculated colorectal carcinoma is necessary for a proper pathological diagnosis. However, due to poor visibility, large pedunculated colorectal carcinomas are difficult to snare and resect en‐bloc using endoscopic resection or polypectomy. Additionally, the bleeding risk of large pedunculated colorectal carcinomas is relatively high. We aimed to assess the feasibility and safety of endoscopic submucosal dissection for large pedunculated colorectal carcinomas.

**Methods:**

We conducted a retrospective multicenter cohort study to assess 36 consecutive patients with 36 large pedunculated colorectal carcinomas who underwent endoscopic submucosal dissection and evaluated the outcomes of endoscopic submucosal dissection. Furthermore, patients were divided into two groups according to the procedure time, and the factors related to the procedure time were assessed.

**Results:**

The mean tumor size was 34.1 ± 9.9 mm. The en‐bloc, complete en‐bloc, and curative resection rates were 97% (35/36), 97% (35/36), and 81% (29/36), respectively. The rate of severe bleeding during the procedure was 11% (4/36); however, it could be controlled endoscopically in all patients. The rate of intraoperative perforation and delayed bleeding was 0% (0/36). Delayed perforations occurred in one patient that required surgery. A long procedure time was correlated with the location of the flexure and poor endoscope operability. No recurrence was observed in any patient. None of the patients died of colorectal carcinoma.

**Conclusions:**

Our results showed the feasibility and safety of endoscopic submucosal dissection for large pedunculated colorectal carcinomas.

## INTRODUCTION

Polypectomy is a standard procedure widely used for the treatment of pedunculated colorectal polyps. Postpolypectomy bleeding (PPB) is one of the most common complications of polypectomy. The risk of PPB after resection of large pedunculated polyps is high.^1^, ^2^, ^3^

Large colorectal polyps have a high risk of submucosal invasive carcinoma.^4^, ^5^ Haggitt et al. stratified the levels of submucosal invasion of pedunculated colorectal carcinomas (CRCs) from 0 to 4 and reported a low risk of metastasis or local recurrence at levels below 4.^6^ Matsuda et al. reported that the incidence of lymph node metastasis was lower in patients with head invasion than in those with stalk invasion.^7^ Therefore, complete en‐bloc resection should be performed for large pedunculated CRCs following an appropriate pathological diagnosis. However, due to poor visibility, large pedunculated CRCs are difficult to snare and resect en bloc using endoscopic mucosal resection (EMR) or polypectomy.

Endoscopic submucosal dissection (ESD) can be performed for complete en‐bloc resection regardless of tumor size and location.^8^ Additionally, ESD has the advantage of being able to visualize submucosal tissue and blood vessels directly and achieve hemostasis in blood vessels during the procedure.

Colorectal ESD is already a commonly performed procedure worldwide, and the feasibility and safety of this procedure have previously been proven.^9^ Recently, several studies have reported the feasibility and safety of ESD for large pedunculated colorectal polyps^10^, ^11^, ^12^, ^13^, ^14^; however, most of these studies were case reports, and the evidence is still insufficient. Therefore, we aimed to assess the feasibility and safety of ESD for large pedunculated CRC and the factors related to procedure time.

## METHODS

### Patients

This was a multicenter, retrospective cohort study comprising an academic hospital and 24 community hospitals with varying levels of experience in colorectal ESD. We performed ESD for 3937 colorectal neoplasms (CRNs) at the Hiroshima GI Endoscopy Research Group between January 2008 and December 2018 (1259 CRNs of retrospective registration between January 2008 and March 2014 and 2678 CRNs of prospective registration between December 2013 and December 2018). Among the CRNs, 36 large pedunculated CRCs were included in the present study and retrospectively analyzed. ESD for a large pedunculated CRC was performed as an alternative treatment to EMR or polypectomy at the endoscopist's discretion for the following three reasons: first, technical difficulty of snaring for en‐bloc resection due to the large tumor size and poor visibility. Second, high risk of bleeding due to the thick stalk, and lastly, the difficulty of prophylactic hemostasis due to the short stalk or poor visibility.

This study was performed in accordance with the ethical standards of the Declaration of Helsinki and its later amendments. The study protocol was reviewed and approved by the institutional review boards of each participating center and registered as UMIN000016197 (Institutional Review Board registration date: January 14, 2015). Written informed consent was obtained from the patients.

### Histopathological assessment

Histopathological assessment was performed according to the 2019 Japanese Society for Cancer of the Colon and Rectum (JSCCR) guidelines^15^ and the World Health Organization classification system.^16^ The pathological diagnosis was Tis carcinoma and submucosal invasive (T1) carcinoma. Complete en‐bloc resection was defined as a pathologically identified en‐bloc resection with negative horizontal and vertical margins. Curative resection was defined based on the 2019 JSCCR curative criteria (negative vertical margin, papillary/tubular adenocarcinoma or medullary carcinoma, negative lymphovascular invasion, submucosal invasion depth <1000 μm, and budding grade 1).^17^ Submucosal invasion depth was assessed according to the Japanese criteria.^15^

### ESD procedure

A 10% glycerin solution containing 0.4% sodium hyaluronate (MucoUp; Johnson & Johnson) and a small amount of indigo carmine (indigo carmine/hyaluronate/glycerol: 0.2/10/10 ml) was used for submucosal injection. DualKnife (Olympus), DualKnife J (Olympus), ITknife nano (Olympus), or SBknife Jr (Sumitomo Bakelite) was used as appropriate for each patient at the endoscopist's discretion. Multiple devices were used, depending on the situation. During a submucosal dissection, prophylactic hemostasis was applied as appropriate while visualizing the blood vessels of the submucosal tissue in the stalk. At the end of the procedure, the exposed vessels on the resected ulcer were coagulated using hemostatic forceps. Hemostatic clips were also used, depending on the situation.

### Evaluation

Clinicopathological characteristics (sex, age, use of antithrombotic agents, tumor location, local difficulty, tumor size, degree of submucosal fibrosis, and pathological diagnosis) and treatment outcomes were assessed. Treatment outcomes; including procedure time, endoscope operability, en‐bloc resection, complete en‐bloc resection, curative resection, bleeding during the procedure, delayed bleeding, intraoperative perforation, delayed perforation, additional surgical resection; operator; and endoscopic device, were evaluated. Moreover, we divided the patients into two groups (long procedure time and short procedure time) according to the procedure time and assessed the factors related to procedure time. Additionally, local or metastatic recurrence, prognosis, characteristics, and treatment outcomes of patients with T1 CRC were examined.

The degree of submucosal fibrosis was classified into three types (F0, no fibrosis; F1, mild fibrosis; and F2, severe fibrosis) based on previous reports.^18^ Poor endoscope operability refers to paradoxical endoscope movement, poor control with adhesions, and passive movements of the lesion or the endoscope resulting from the patient's respiration or heartbeats.^19^ Severe bleeding was defined as bleeding that required multiple coagulation (≥ 10 times) during the procedure.^20^ Delayed bleeding was defined as ≥ 2 g/dl decrease in hemoglobin levels compared to the preoperative level, apparent bleeding, or massive melena after the procedure.^21^ Experts were defined as endoscopists who had performed more than 80 cases of colorectal ESD.^22^The procedure time of ESD was defined as the time from the beginning of the submucosal injection until the complete resection of the lesion.

### Statistical analysis

Quantitative data are presented as the mean ± standard deviation or percentage. Continuous variables were analyzed using Student's t‐test. Categorical variables were analyzed using Fisher's exact test or the chi‐squared test. Statistical significance was set at *p* < 0.05. The JMP statistical software (version 15.0.0; SAS Institute) was used for all statistical analyses.

## RESULTS

### Clinicopathological characteristics of the enrolled patients

Table 1 shows the clinicopathological characteristics of the enrolled patients and their tumors. The tumors were most often located in the left colon (56% [20/36]). The rate of tumors located at the flexure was 11% (4/36), and the rate of tumors straddling a fold was 28% (10/36). The mean tumor size was 34.1 ± 9.9 mm. The rate of T1 CRC was 25% (9/36). Four (11%) patients had head invasion, none had submucosal invasion <1000 μm and five (14%) had submucosal invasion ≥1000 μm.

**TABLE 1:** Clinicopathological characteristics.

Variables	*n* = 36
Sex, male	32 (89)
Age, mean ± SD, years	63.9 ± 11.6
Use of antithrombotic agents	6 (17)
Tumor location	
Right colon	9 (25)
Left colon	20 (56)
Rectum	7 (19)
Local difficulty	
Flexure	4 (11)
Straddling a fold	10 (28)
Tumor size, mean ± SD, mm	34.1 ± 9.9
SM fibrosis	
None/mild	34 (94)
Severe	2 (6)
Pathological diagnosis	
Tis carcinoma	27 (75)
T1 carcinoma, SM depth (μm)	
Head invasion	4 (11)
SM < 1000	0 (0)
SM ≥1000	5 (14)

SD, standard deviation; SM, submucosal (%)

### ESD outcomes

Table 2 lists ESD outcomes. The mean procedure time was 49.9 ± 39.1 min. The en bloc, complete en bloc, and curative resection rates were 97% (35/36), 97% (35/36), and 81% (29/36), respectively. The poor endoscope operability rate was 33% (12/36). The severe bleeding rate during the procedure was 11% (4/36); however, it was endoscopically controlled in all patients. No patient experienced delayed bleeding. Delayed perforations occurred in one patient who required surgery. Needle‐type knives were used most often, and multiple devices were used in some patients. Any traction technique was not used in conjunction with ESD.

**TABLE 2:** Endoscopic submucosal dissection outcomes.

Variables	*n* = 36
Procedure time, mean ± SD, min	49.9 ± 39.1
Endoscope operability	
Good	24 (67)
Poor	12 (33)
En bloc resection	35 (97)
Complete en‐bloc resection	35 (97)
Curative resection	29 (81)
Bleeding during procedure	
None/mild	32 (89)
Severe	4 (11)
Delayed bleeding	0 (0)
Intraoperative perforation	0 (0)
Delayed perforation	1 (3)
Closure of post‐ESD ulcer	7 (19)
Additional surgical resection	7 (19)
Operator	
Expert	32 (89)
Non‐expert	4 (11)
Endoscopic device^*^	
DualKnife/DualKnife J	29 (81)
ITknife nano	10 (28)
SBknife Jr	10 (28)

SD, standard deviation.

^*^Including duplicate patients (%)

### Factors related to procedure time

The median procedure time was 40 min. The lesions were divided into two groups based on the median procedure time: long (<40 min) and short (≥40 min). The short and long procedure time groups comprised 20 and 16 patients, respectively. Tables 3 and 4 display a comparison of clinicopathological characteristics and ESD outcomes between the two groups. The rate of tumors located at the flexure was significantly higher in the long procedure time group (25%, 4/16) than in the short procedure time group (0%, 0/20; *p* = 0.0309). The mean tumor size was larger in the long procedure time group (37.5 ± 10.3 mm) than in the short procedure time group (31.4 ± 8.9 mm), but there was no significant difference in the mean tumor size between the two groups (*p* = 0.0659). The rate of poor endoscope operability was significantly higher in the long procedure time group (62%, 10/16) than in the short procedure time group (10%, 2/20; *p* = 0.0014). There were no significant differences in other clinicopathological characteristics or ESD outcomes between the two groups.

**TABLE 3:** Clinicopathological characteristics related to procedure time.

	Procedure time		
Variables	short *n* = 20	Long *n* = 16	*p*‐value
Sex, male	16 (80)	16 (100)	0.1131
Age, mean ± SD, years	67.1 ± 8.1	60.0 ± 14.3	0.0681
Use of antithrombotic agents	2 (10)	4 (25)	0.3738
Tumor location			
Right colon	3 (15)	6 (38)	0.2963
Left colon	14 (70)	8 (50)	
Rectum	3 (15)	2 (12)	
Local difficulty			
Flexure	0 (0)	4 (25)	0.0309
Straddling a fold	5 (25)	5 (31)	0.7225
Tumor size, mean ± SD, mm	31.4 ± 8.9	37.5 ± 10.3	0.0659
SM fibrosis			
None/mild	20 (100)	14 (88)	0.1905
Severe	0 (0)	2 (12)	
Pathological diagnosis			
Tis carcinoma	15 (75)	12 (76)	0.9798
T1 carcinoma, SM depth (μm)			
Head invasion	2 (10)	2 (12)	
SM <1000	0 (0)	0 (0)	
SM ≥1000	3 (15)	2 (12)	

SD, Standard deviation; SM, submucosal (%)

**TABLE 4:** Endoscopic submucosal dissection outcomes related to procedure time.

	Procedure time		
Variables	short *n* = 20	long *n* = 16	*p*‐value
Endoscope operability			
Good	18 (90)	6 (38)	0.0014
Poor	2 (10)	10 (62)	
En‐bloc resection	20 (100)	15 (94)	0.4444
Complete en‐bloc resection	20 (100)	15 (94)	0.4444
Curative resection	16 (80)	13 (81)	1
Bleeding during procedure			
None/mild	18 (90)	14 (88)	1
Severe	2 (10)	2 (12)	
Delayed bleeding	0 (0)	0 (0)	1
Intraoperative perforation	0 (0)	0 (0)	1
Delayed perforation	0 (0)	1 (6)	0.4444
Closure of post‐ESD ulcer	2 (10)	5 (31)	0.2036
Additional surgical resection	4 (20)	3 (19)	1
Operator			
Expert	19 (95)	13 (81)	0.3033
Non‐expert	1 (5)	3 (19)	
Endoscopic device^*^			
DualKnife/DualKnife J	15 (75)	14 (88)	0.4264
ITknife nano	4 (20)	6 (38)	0.2853
SBknife Jr	5 (25)	5 (31)	0.7225

ESD, endoscopic submucosal dissection; SD, standard deviation.

^*^Including duplicate patients (%)

### Characteristics of large pedunculated T1 CRC

Characteristics of the nine patients with T1 CRC are shown in Table 5. Of the nine patients, four had head invasions, and five had submucosal invasions of ≥1000 μm. Most tumors were located in the sigmoid colon (89%, 8/9). The minimum and maximum tumor sizes were 20 and 50 mm, respectively. The en bloc and complete en‐bloc resection rates were 100% (9/9). The curative resection rate was 33% (3/9 patients). Five patients showed lymphovascular invasion. One patient had a tumor budding grade 2. One patient had a mucinous histology. Additional surgical resection was performed in seven patients. In patient 3, an additional surgical resection was performed because of mucinous histology in the invasive portion. No local or metastatic recurrence was observed in any patient. None of the patients died of CRC (observation period: 47 months).

**TABLE 5:** Characteristics of large pedunculated colorectal T1 carcinoma.

Patient	Sex	Age (years)	Use of antithrombotic agents	Tumor location	Local difficulty	Tumor size	Histology	SM depth (μm)	Ly/V	Budding grade	SM fibrosis	Procedure time	Endoscope operability	En bloc resection	Complete en bloc resection	Curative resection	Bleeding during procedure	Intraoperative/delayed perforation	Delayed bleeding	Closure of post‐ESD ulcer	Additional surgical resection	Operator	Endoscopic device
1	Male	65	Aspirin	S/C	Flexure	50	muc	5000	+/‐	2	mild	110	Poor	Yes	Yes	No	None/mild	None	None	No	Yes	expert	DualKnife J, ITknife nano
2	Male	77	None	S/C	None	25	tub	head invasion	‐/‐	1	mild	141	Good	Yes	Yes	Yes	Severe	None	None	No	No	expert	SBknife Jr
3	Male	30	None	S/C	Straddling a fold	40	tub	head invasion	‐/‐	1	mild	150	Poor	Yes	Yes	Yes	None/mild	None	None	Yes	Yes	expert	DualKnife J, ITknife nano
4	Male	68	None	S/C	None	30	tub	11000	‐/+	1	None	15	Good	Yes	Yes	No	None/mild	None	None	No	Yes	expert	DualKnife J
5	Male	68	None	S/C	None	30	tub	7000	‐/‐	1	None	15	Good	Yes	Yes	No	None/mild	None	None	No	Yes	expert	DualKnife J
6	Male	56	None	S/C	None	50	tub	5000	+/+	1	mild	95	Poor	Yes	Yes	No	None/mild	None	None	No	Yes	expert	DualKnife J, ITknife nano
7	Male	66	None	S/C	Straddling a fold	25	tub	Head invasion	‐/‐	1	None	29	Good	Yes	Yes	Yes	None/mild	None	None	No	No	expert	DualKnife J
8	Male	53	None	RS	None	20	tub	3000	+/‐	1	None	30	Good	Yes	Yes	No	None/mild	None	None	No	Yes	expert	DualKnife J
9	Male	75	None	S/C	None	25	tub	head invasion	‐/+	1	None	10	Good	Yes	Yes	No	None/mild	None	None	No	Yes	expert	DualKnife J, ITknife nano

Abbreviations: Ly/V, lymphatic invasion/venous invasion; muc, mucinous adenocarcinoma; S/C, sigmoid colon; SM, submucosal; tub, tubular adenocarcinoma.

## DISCUSSION

Our data showed that the outcomes of ESD for large pedunculated CRCs were good, without severe complications. As mentioned previously, the colorectal ESD procedure is well established.^9^ There have been several studies on ESD for large pedunculated colorectal polyps.^10^, ^11^, ^12^, ^13^, ^14^ Recently, a multicenter retrospective study was reported^14^; however, most previous studies were conducted on a small number of patients, and the evidence is still insufficient. Our results are consistent with those of previous studies. Additionally, we assessed factors related to procedure time. We found that a long procedure time was correlated with the location at the flexure and poor endoscope operability. To our knowledge, this is the first study to discuss the factors related to ESD procedure time for large pedunculated CRC. Our results demonstrated the feasibility and safety of ESD for large pedunculated CRC.

We previously assessed the long‐term outcomes for pedunculated T1 CRCs after endoscopic resection or surgical resection and reported that the risk of lymph node metastasis, based on the JSCCR diagnostic criteria, should be evaluated after endoscopic resection for pedunculated T1 CRC.^23^ In addition, additional surgery should be considered in patients at high risk of lymph node metastasis.^23^ Therefore, complete en‐bloc resection should be performed for large pedunculated CRC, and appropriate pathological diagnosis should be performed to assess the risk of lymph node metastasis. However, due to poor visibility, large pedunculated CRCs are difficult to snare and resect en bloc using EMR or polypectomy.

An ESD can be performed for complete en‐bloc resection regardless of the tumor size and location.^8^ Choi et al.^10^ retrospectively compared the feasibility of ESD and polypectomy for giant pedunculated polyps and reported that the en‐bloc resection rate was 100% (23/23) in the ESD group and 90% (18/20) in the polypectomy group regardless of whether the endoscopists judged that it could be possible. In this study, the rates of en‐bloc resection and complete en‐bloc resection were 97% (35/36) each, similar to that reported previously.^10^, ^14^ The rates of en‐bloc resection and complete en‐bloc resection for T1 CRC were 100% (9/9), which was also good. Additionally, there was no local or metastatic recurrence in any of the patients, and their prognoses were good. Furthermore, complete en‐bloc resection using ESD was possible for large pedunculated CRCs, including T1 CRC. Appropriate pathological diagnosis and evaluation of the risk of lymph node metastasis were also possible. In ESD, submucosal dissection is performed while visualizing the submucosal layer. Therefore, even in cases of stalk invasion, the tumor could be removed without tumor remnant by visualizing the tumor mass and dissecting the submucosal layer under the tumor. Therefore, our results showed that ESD is an effective treatment for large pedunculated CRC regarding complete en‐bloc resection.

PPB is one of the most common complications of polypectomy. The risk of PPB after resection of large pedunculated polyps is high because large feeding vessels traverse the stalk to supply the head of the polyp.^1^ Soh et al.^3^ reported that polyp size ≥ 20 mm and stalk diameter ≥ 4 mm increased the risk of immediate PPB after resectioning large pedunculated polyps. In endoscopic resection of pedunculated polyps, especially in cases with large tumor sizes, it is necessary to manage bleeding with effective preventive methods.

Injection (adrenaline and epinephrine)^24^ and mechanical (endoloop and hemostatic clips)^3^, ^25^, ^26^ therapies have been developed as bleeding prevention methods after polypectomy for pedunculated polyps, and their efficacies have been reported. However, the application of an endoloop can be challenging, particularly for polyps with a large head or short stalk, because endoloops have a lower expansible force and stiffness. Additionally, prophylactic hemostatic clips might not completely cover the stalk of large pedunculated polyps and stop blood flow in the stalks completely when the stalk is thick. On the other hand, ESD has the advantage of being able to visualize submucosal tissue and blood vessels directly and apply hemostasis to blood vessels during the procedure. Choi et al.^10^ reported that the rate of intraoperative bleeding requiring endoscopic hemostasis was higher with polypectomy (15%, 3/20) than with ESD (4.3%, 1/23). Additionally, they reported no cases of delayed bleeding after ESD (0%, 0/23). Chiba et al.^14^ evaluated the feasibility of ESD for large pedunculated polyps with wide stalks in a retrospective multicenter study and reported no cases of delayed bleeding after ESD (0%, 0/29). In our study, there were no patients with delayed bleeding after ESD (0%, 0/36), similar to previous studies.^10^, ^14^ In contrast, the rate of severe intraoperative bleeding was 11% (4/36 patients). This result might be due to the large tumor size (mean, 34.1 mm), thick stalk, and the presence of vessels. However, it could be controlled endoscopically in all patients. Therefore, our results show that ESD is an effective treatment for large pedunculated CRC in terms of bleeding because prophylactic hemostasis could be applied as appropriate while visualizing the blood vessels of the submucosal tissue in the stalk during a submucosal dissection.

In this study, the mean procedure time was 49.9 ± 39.1 min, and the median procedure time was 40 min, similar to the previous study.^10^ In this study, the rate of tumors located at the flexure and poor endoscope operability were significantly higher in the long procedure time group than in the short procedure time group. In the long procedure time group, delayed perforation occurred in one patient. The polyp was a 50 mm‐sized Tis carcinoma located at the flexure of the sigmoid colon. Bleeding during the procedure was severe, and the muscle layer was severely in traction. This could have caused the delayed perforation. In such cases, endoscopic closure of the ulcers may be effective in preventing delayed perforation. Poor endoscope operability during ESD was reported as a significant independent predictor of perforation.^19^ Lesion located at the colonic flexures was reported to be a strong indicator of longer procedure time and a significant risk factor for the incidence of perforation.^27^ Our results were consistent with the previous reports.^19^, ^27^ However, the outcomes were equally good in the long procedure time group. Our results showed that ESD is an effective treatment for large pedunculated CRC, even in difficult cases. There is a possibility that ESD for large pedunculated CRC could be performed more safely and effectively using different devices (single‐balloon overtubes,^28^ scissors‐type knives,^29^, ^30^ insulated‐tip knives, etc.), depending on the size and situation. However, ESD has cost, hospitalization, and procedure time disadvantages and should be performed in selected cases where EMR or polypectomy is difficult.

Our study had some limitations. First, it was a multicenter, retrospective cohort study; however, the sample size was relatively small. A prospective study with a larger number of patients should be conducted in the near future to confirm our results. Second, we lacked information on the thickness and length of the stalks and were, therefore, unable to evaluate them. Future studies should consider not only the tumor size but also the thickness and length of the stalk. Third, we lacked a control group and were unable to compare ESD with EMR and polypectomy. Forth, ESD was selected as an alternative treatment to EMR or polypectomy at the endoscopist's discretion, and selection bias exists.

In conclusion, ESD outcomes for large pedunculated CRC were good and without severe complications. Our results show that ESD is an effective and safe treatment for large pedunculated CRC.

## CONFLICT OF INTEREST STATEMENT

The authors declared ST is the president of JGES.

## DATA ACCESSIBILITY STATEMENT

All the data used to support the findings of this study are included in this article.

## References

[1] Isohata N , Nemoto D , Utano K *et al.* Morphometric study of the blood supply of pedunculated colon polyps: What is the optimal position on the stalk for snare resection? Endosc Int Open 2015; 3: E655–8. 26716131 10.1055/s-0034-1393082 PMC4683148

[2] Dobrowolski S , Dobosz M , Babicki A , Głowacki J , Nałęcz A . Blood supply of colorectal polyps correlates with risk of bleeding after colonoscopic polypectomy. Gastrointest Endosc 2006; 63: 1004–9. 16733117 10.1016/j.gie.2005.11.063

[3] Soh JS , Seo M , Kim KJ . Prophylactic clip application for large pedunculated polyps before snare polypectomy may decrease immediate postpolypectomy bleeding. BMC Gastroenterol 2020; 20: 68. 32164613 10.1186/s12876-020-01210-5 PMC7069010

[4] O'Brien MJ , Winawer SJ , Zauber AG *et al.* The National Polyp Study. Patient and polyp characteristics associated with high‐grade dysplasia in colorectal adenomas. Gastroenterology 1990; 98: 371–9. 2403953

[5] Ahlawat SK , Gupta N , Benjamin SB , Al‐Kawas FH . Large colorectal polyps: Endoscopic management and rate of malignancy: Does size matter? J Clin Gastroenterol 2011; 45: 347–54. 20871408 10.1097/MCG.0b013e3181f3a2e0

[6] Haggitt RC , Glotzbach RE , Soffer EE , Wruble LD . Prognostic factors in colorectal carcinomas arising in adenomas: Implications for lesions removed by endoscopic polypectomy. Gastroenterology 1985; 89: 328–36. 4007423 10.1016/0016-5085(85)90333-6

[7] Matsuda T , Fukuzawa M , Uraoka T *et al.* Risk of lymph node metastasis in patients with pedunculated type early invasive colorectal cancer: A retrospective multicenter study. Cancer Sci 2011; 102: 1693–7. 21627735 10.1111/j.1349-7006.2011.01997.x PMC11159017

[8] Tanaka S , Terasaki M , Kanao H , Oka S , Chayama K . Current status and future perspectives of endoscopic submucosal dissection for colorectal tumors. Dig Endosc 2012; 24: 73–9. 22533757 10.1111/j.1443-1661.2012.01252.x

[9] Boda K , Oka S , Tanaka S *et al.* Clinical outcomes of endoscopic submucosal dissection for colorectal tumors: A large multicenter retrospective study from the Hiroshima GI Endoscopy Research Group. Gastrointest Endosc 2018; 87: 714–22. 28623057 10.1016/j.gie.2017.05.051

[10] Choi YS , Lee JB , Lee EJ *et al.* Can endoscopic submucosal dissection technique be an alternative treatment option for a difficult giant (≥30 mm) pedunculated colorectal polyp? Dis Colon Rectum 2013; 56: 660–6. 23575407 10.1097/DCR.0b013e318276d2b9

[11] Yang CW , Yen HH , Chen YY , Soon MS . Use of dual knife for large pedunculated colorectal polyps. Surg Laparosc Endosc Percutan Tech 2014; 24: 444–7. 25198067 10.1097/SLE.0000000000000097

[12] Ma L , Zhai Y , Chai N *et al.* Insulated‐tip knife endoscopic polypectomy for difficult pedunculated colorectal polyps: A prospective pilot study. Int J Colorectal Dis 2017; 32: 287–90. 27987015 10.1007/s00384-016-2699-y

[13] Jawaid S , Draganov PV , Yang D . Endoscopic resection of large pedunculated colon polyps using only a scissor‐type knife: A case series. VideoGIE 2020; 5: 264–6. 32529165 10.1016/j.vgie.2020.02.016 PMC7277036

[14] Chiba H , Tachikawa J , Arimoto J *et al.* Endoscopic submucosal dissection of large pedunculated polyps with wide stalks: A retrospective multicenter study. Endoscopy 2021; 53: 77–80. 32645728 10.1055/a-1194-4413

[15] Matsumoto A , Tanaka S , Oba S *et al.* Outcome of endoscopic submucosal dissection for colorectal tumors accompanied by fibrosis. Scand J Gastroenterol 2010; 45: 1329–37. 20626303 10.3109/00365521.2010.495416

[16] Hayashi N , Tanaka S , Nishiyama S *et al.* Predictors of incomplete resection and perforation associated with endoscopic submucosal dissection for colorectal tumors. Gastrointest Endosc 2014; 79: 427–35. 24210654 10.1016/j.gie.2013.09.014

[17] Higashiyama Y , Oka S , Tanaka S *et al.* Risk factors for bleeding after endoscopic submucosal dissection of gastric epithelial neoplasm. Dig Endosc 2011; 23; 290–5. 21951088 10.1111/j.1443-1661.2011.01151.x

[18] Tajiri H , Kitano S . Complications associated with endoscopic mucosal resection: Definition of bleeding that can be viewed as accidental. Dig Endosc 2004; 16: S134–6.

[19] Pimentel‐Nunes P , Dinis‐Ribeiro M , Ponchon T *et al.* Endoscopic submucosal dissection: European Society of Gastrointestinal Endoscopy (ESGE) Guideline. Endoscopy 2015; 47: 829–54. 26317585 10.1055/s-0034-1392882

[20] Japanese Society for Cancer of the Colon and Rectum . Japanese classification of colorectal, appendiceal, and anal carcinoma: The 3d English edition [secondary publication]. J Anus Rectum Colon 2019; 3: 175–95. 31768468 10.23922/jarc.2019-018 PMC6845287

[21] Hamilton SR , Boffetta P , Ilyas M *et al.* Tumours of the colon and rectum. In: Bosman FT , Carneiro F , Hruban RH , Theise ND (eds), WHO Classification of Tumours of the Digestive System, 4th edn, IARC Press, Lyon 2010:103–42.

[22] Hashiguchi Y , Muro K , Saito Y *et al.* Japanese Society for Cancer of the Colon and Rectum (JSCCR) Guidelines 2019 for the treatment of colorectal cancer. Int J Clin Oncol 2020; 25: 1–42. 31203527 10.1007/s10147-019-01485-z PMC6946738

[23] Asayama N , Oka S , Tanaka S *et al.* Long‐term outcomes after treatment for pedunculated‐type T1 colorectal carcinoma: A multicenter retrospective cohort study. J Gastroenterol 2016; 51: 702–10. 26573300 10.1007/s00535-015-1144-2

[24] Giorgio PD , Luca LD , Calcagno G , Rivellini G , Mandato M , De Luca B . Detachable snare versus epinephrine injection in the prevention of postpolypectomy bleeding: A randomized and controlled study. Endoscopy 2004; 36: 860–3. 15452780 10.1055/s-2004-825801

[25] Gweon TG , Lee KM , Lee SW *et al.* Effect of prophylactic clip application for the prevention of postpolypectomy bleeding of large pedunculated colonic polyps: A randomized controlled trial. Gastrointest Endosc 2021; 94: 148–54. 33417897 10.1016/j.gie.2020.12.040

[26] Brandimarte G , Tursi A . Endoscopic snare excision of large pedunculated colorectal polyps: A new, safe, and effective technique. Endoscopy 2001; 33: 854–7. 11571681 10.1055/s-2001-17329

[27] Hori K , Uraoka T , Harada K *et al.* Predictive factors for technically difficult endoscopic submucosal dissection in the colorectum. Endoscopy 2014; 46: 862–70. 25208032 10.1055/s-0034-1377205

[28] Asayama N , Oka S , Tanaka S *et al.* Clinical usefulness of a single‐use splinting tube for poor endoscope operability in deep colonic endoscopic submucosal dissection. Endosc Int Open 2016; 4: E614–7. 27556066 10.1055/s-0042-105434 PMC4993897

[29] Kuwai T , Yamaguchi T , Imagawa H *et al.* Endoscopic submucosal dissection of early colorectal neoplasms with a monopolar scissor‐type knife: Short‐ to long‐term outcomes. Endoscopy 2017; 49: 913–8. 28743145 10.1055/s-0043-113631

[30] Oka S , Tanaka S , Takata S , Kanao H , Chayama K . Usefulness and safety of SB knife in endoscopic submucosal dissection for colorectal tumors. Dig Endosc 2012; 24: 90–5. 22533760 10.1111/j.1443-1661.2012.01255.x

